# Supplementary material for: Development of a VLP-Based Vaccine Displaying an xCT Extracellular Domain for the Treatment of Metastatic Breast Cancer
Source: Cancers (Basel). 2020 Jun 8;12(6):1492. doi: 10.3390/cancers12061492 (PMC7352461; doi:10.3390/cancers12061492)
Supplement: Supplementary file 1 [file cancers-12-01492-s001.pdf]

cancers

Supplementary Materials:

Development of a VLP-Based Vaccine Displaying an xCT Extracellular Domain for the Treatment of Metastatic Breast Cancer

Valeria Rolih, Jerri Caldeira, Elisabetta Bolli, Ahmad Salameh, Laura Conti, Giuseppina Barutello, Federica Riccardo, Jolanda Magri, Alessia Lamolinara, Karla Parra, Paloma Valenzuela, Giulio Francia, Manuela Iezzi, Federica Pericle and Federica Cavallo

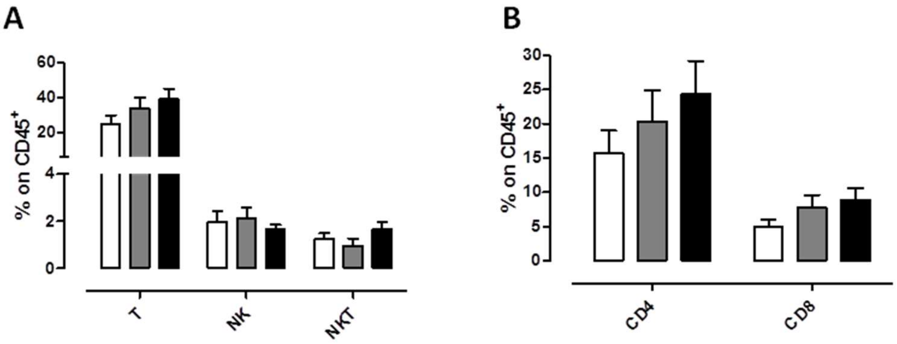

Figure S1. FACS analysis of the immune infiltrate of tumors derived from AX09 (black bars) or MS2 wt (grey bars) immunized or untreated (white bars) BALB/c mice challenged s.c. with 4T1 tumorspheres. (**A**) Percentage ± SEM of CD45+ cells expressing the markers of T lymphocytes (CD3+CD49−), NK cells (CD3−CD49+), NKT cells (CD3−CD49+). (**B**) Percentage ± SEM of CD4+ or CD8+ T cells gated on CD45+ cells.
